# Temporal Profile of Pneumonia After Stroke

**DOI:** 10.1161/STROKEAHA.120.032787

**Published:** 2021-09-14

**Authors:** Jeroen C. de Jonge, Diederik van de Beek, Patrick Lyden, Marian C. Brady, Philip M. Bath, H. Bart van der Worp

**Affiliations:** Department of Neurology and Neurosurgery, UMC Utrecht Brain Center, University Medical Center Utrecht, Utrecht University, the Netherlands (J.C.d.J., H.B.v.d.W.).; Department of Neurology, Amsterdam University Medical Center, Amsterdam Neuroscience, the Netherlands (D.v.d.B.).; Departments of Physiology and Neuroscience and Neurology, USC Keck School of Medicine, Los Angeles, CA (P.L.).; Nursing, Midwifery and Allied Health Professions Research Unit, Glasgow Caledonian University, United Kingdom (M.C.B.).; Stroke Trials Unit, Division of Clinical Neuroscience, School of Medicine, University of Nottingham, United Kingdom (P.M.B.).

**Keywords:** death, pneumonia, stroke

## Abstract

The occurrence of pneumonia after stroke is associated with a higher risk of poor outcome or death. We assessed the temporal profile of pneumonia after stroke and its association with poor outcome at several time points to identify the most optimal period for testing pneumonia prevention strategies.

**Methods:**

We analyzed individual patient data stored in the VISTA (Virtual International Stroke Trials Archive) from randomized acute stroke trials with an inclusion window up to 24 hours after stroke onset and assessed the occurrence of pneumonia in the first 90 days after stroke. Adjusted odds ratios and hazard ratios were calculated for the association between pneumonia and poor outcome and death by means of logistic and Cox proportional hazard regression, respectively, at different times of follow-up.

**Results:**

Of 10 821 patients, 1017 (9.4%) had a total of 1076 pneumonias. Six hundred eighty-nine (64.0%) pneumonias occurred in the first week after stroke. The peak incidence was on the third day and the median time of onset was 4.0 days after stroke (interquartile range, 2–12). The presence of a pneumonia was associated with an increased risk of poor outcome (adjusted odds ratio, 4.8 [95% CI, 3.8–6.1]) or death (adjusted hazard ratio, 4.1 [95% CI, 3.7–4.6]). These associations were present throughout the 90 days of follow-up.

**Conclusions:**

Two out of 3 pneumonias in the first 3 months after stroke occur in the first week, with a peak incidence on the third day. The most optimal period to assess pneumonia prevention strategies is the first 4 days after stroke. However, pneumonia occurring later was also associated with poor functional outcome or death.

## Abstract 2

Supplemental Digital Content is available in the text.

About 30% of patients develop an infection in the first days after stroke, of which about one-third is a pneumonia.^1^ The development of an infection after stroke is associated with a higher risk of poor outcome or death.^1–4^ In particular, pneumonia contributes to early mortality after stroke, and it has been estimated that 10% of the deaths within 30 days after stroke are attributable to pneumonia.^5,6^

Limited data from small patients groups suggest that most pneumonias occur in the first 48 to 72 hours after stroke, but these studies only evaluated the development of pneumonia during hospital admission or within the first week or month after stroke.^7–10^ One study of 369 patients observed 2 peaks in the incidence of pneumonia after stroke: one peak at admission and one peak at day 3 or 4.^11^ Most of the other previous studies made no further specification on the temporal profile of pneumonia after stroke and this has therefore remained largely unknown.

Greater knowledge about the temporal profile of the relation between pneumonia and functional outcome or death is important to design more effective prevention strategies.^12^ Knowledge of this temporal profile may identify the most optimal period for (antibiotic) prevention of pneumonia after stroke. In 2 large clinical trials on the prevention of infections after stroke, antibiotic treatment was started within 24 or 48 hours of stroke onset, for a period of 4 or 7 days.^13,14^ These trials failed to demonstrate a benefit of preventive antibiotics on the occurrence of pneumonia or on functional outcome. Our objective is to assess the temporal profile of pneumonia in a large number of patients with acute stroke and its relationship with poor outcome or death at different time points.

## Methods

We conducted a retrospective analysis of anonymized prospectively collected individual patient data from the acute ischemic stroke or intracerebral hemorrhage databases of the VISTA (Virtual International Stroke Trials Archive). VISTA collects anonymized data from completed randomized stroke trials; its design has been reported previously.^15^ All included studies had individual ethics approval, and all participants gave consent. The data that support the findings of this study are available from the corresponding author upon reasonable request. A completed PRISMA-IPD checklist (Preferred Reporting Items for Systematic Review and Meta-Analyses of Individual Patient Data) is added as Data Supplement.

To assess the occurrence of pneumonia early after stroke onset, we included patient data from trials with an inclusion window up to 24 hours after stroke onset; that reported on the occurrence of pneumonia; and had a follow-up period of 90 days. We expected that all studies would report on the frequency of pneumonia because most pneumonias fulfill the criteria for a serious adverse event (SAE). SAEs that include the term pneumonia or lung infection were classified as pneumonia (Appendix I in the Data Supplement). The date of onset of pneumonia was defined as the start date of the relevant SAE reported in the adverse event forms of the trials. Pneumonia recorded within 7 days following a previously documented pneumonia was considered as a single pneumonia. We assessed the temporal profile of the occurrence of pneumonia in the 90 days after stroke in the complete patient population, as well as in subgroups based on age (≤60 versus >60 years), sex, stroke type (ischemic stroke versus intracerebral hemorrhage), baseline score on the National Institutes of Health Stroke Scale (NIHSS; NIHSS score ≤12 versus >12), and treatment with alteplase. To gain more insight into the temporal profile of pneumonia after stroke, we compared it with that of urinary tract infection (UTI). Also, we assessed the relation between pneumonia after stroke and death or poor functional outcome (defined as a score of ≥3 on the modified Rankin Scale after 90) for all pneumonias combined as well as for pneumonias that occurred in specific time periods (days 0–6, days 7–30, days 31–60, and days 61–90). Finally, we assessed the time from reported pneumonia onset to death in patients who died and compared this time between pneumonias on days 0 to 6 and those on days 7 to 90. Other variables of interest included time from stroke onset to start of trial treatment, smoking, and comorbidities (history of diabetes, atrial fibrillation, hypertension, coronary heart disease, heart failure, myocardial infarction, previous stroke or transient ischemic attack, and hypocholesteremia).

### Statistical Analysis

The absolute and relative risks of pneumonia or UTI and the median time from stroke onset to onset of pneumonia or UTI were summarized for the first 90 days after stroke. The same numbers were calculated for the first 7 days (day 0–6). The start day of pneumonia was compared in subgroups by means of an independent *t* test or Mann-Whitney *U* test, where appropriate. Associations between baseline characteristics and the development of pneumonia in the 90 days after stroke were assessed by univariate analysis (χ^2^ analysis, independent *t* test or Mann-Whitney test, where appropriate). Variables with the univariate *P*<0.15 were selected for inclusion in a multivariable logistic regression to identify independent predictors of pneumonia. A crude hazard ratio and adjusted hazard ratio with corresponding 95% CI were calculated for the risk of death after pneumonia by means of a Cox proportional hazard model with time-dependent covariates. Crude odds ratios (OR) and adjusted ORs (aOR) with corresponding 95% CIs were calculated for the association between pneumonia and poor outcome by means of logistic regression. In addition, aORs were calculated for (1) the occurrence of pneumonia in specific time periods (days 0–6, days 7–30, days 31–60, and days 61–90) and (2) the date of onset of pneumonia as a continuous variable, in relation to poor outcome and death. The aORs and adjusted hazard ratios were adjusted for factors known to be related to outcome after stroke: age, sex, stroke severity (defined as the score on the NIHSS), stroke type, hypertension, diabetes, myocardial infarction, and treatment with alteplase. Statistical significance was set at 95%. Adjustments for multiplicity of testing were not performed. All data were analyzed using SPSS version 25 (IBM, Chicago, IL).

## Results

Our study included 10 821 patients from both the treatment as placebo group from 9 neutral randomized clinical trials conducted between 1995 and 2013. Table 1 summarizes the baseline characteristics. The mean age was 71 years (interquartile range [IQR], 61–78), 44.3% were female, and the majority of patients had ischemic stroke (89.2%) rather than intracerebral hemorrhage. Since the studies were performed in 1995 and 2013, none of the patients received mechanical thrombectomy. The median NIHSS score was 12: the study consisted of 234 minor stroke (NIHSS score 0–4), 7017 moderate stroke (NIHSS score 5–15), 2317 moderate to severe stroke (NIHSS score 16–20), and 1251 severe stroke (NIHSS score 21–42).

**Table 1.**
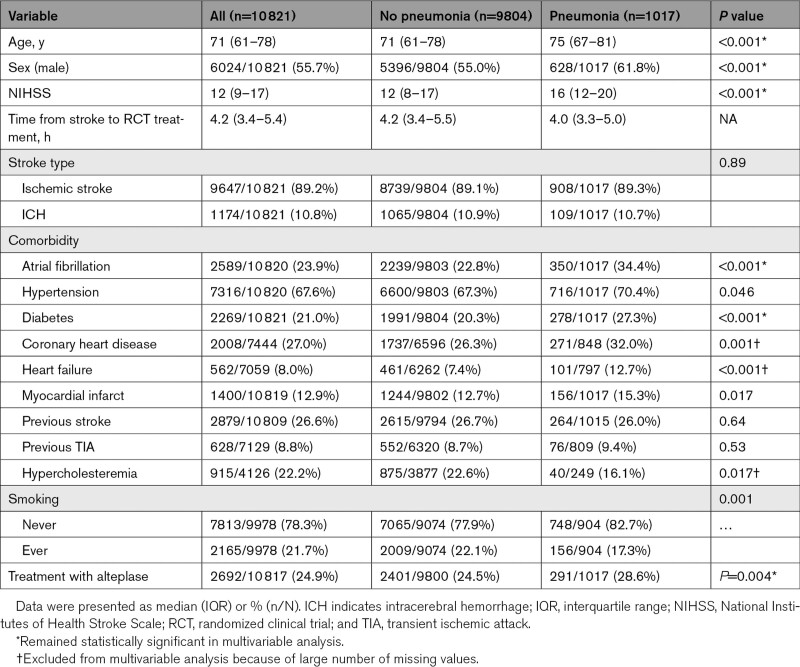
Baseline Characteristics

### Infection Incidence and Temporal Profile

**Figure 1.**
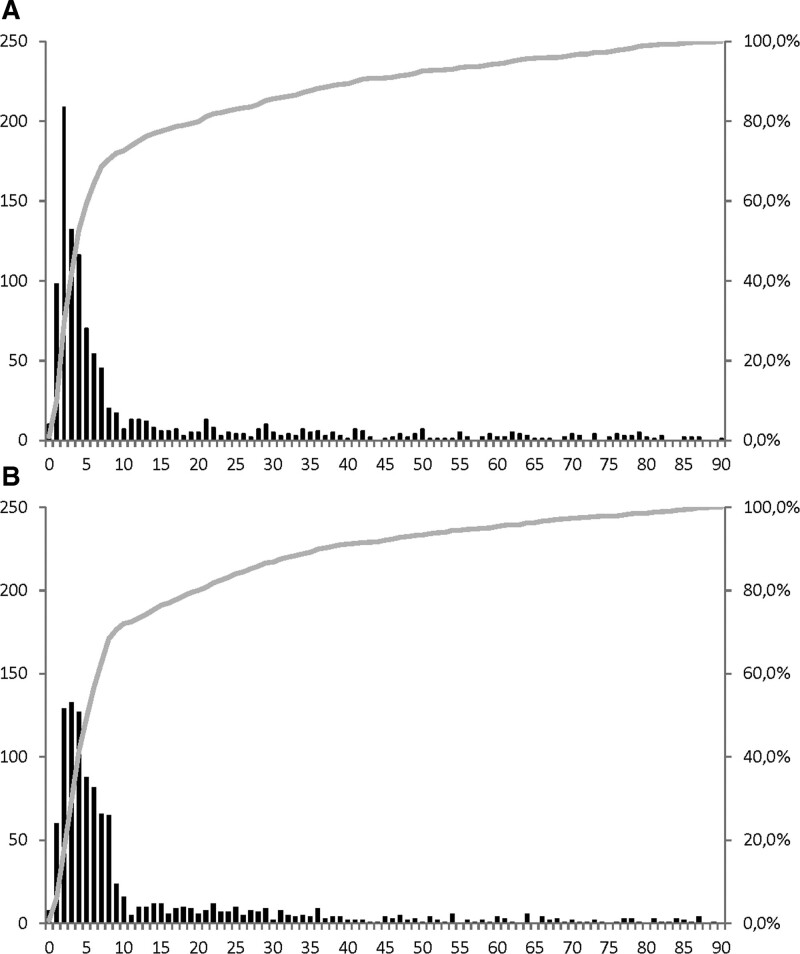
**Temporal profile of post-pneumonia and urinary tract infection.** Absolute number (black bars) and cumulative percentage (gray line) of pneumonia (**A**) and urinary tract infection (**B**) in the first 90 d after stroke.

**Figure 2.**
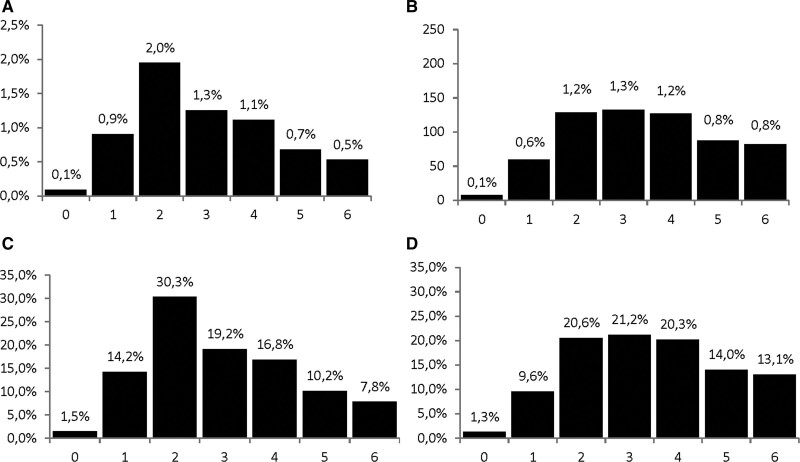
**Risk of post-pneumonia and urinary tract infection.** Absolute risk of pneumonia (**A**) and urinary tract infection (**B**) in the first 7 d after stroke. Relative risk of pneumonia (**C**) and urinary tract infections (**D**), defined as the number of pneumonia or urinary tract infections on a specific day in relation to all pneumonia or urinary tract infections in the first 7 d after stroke.

Thirty-seven pneumonias recorded within 7 days following a previously documented pneumonia were considered as a single pneumonia. A total of 1017 patients had a total of 1076 pneumonias in the first 90 days after stroke (cumulative incidence of 9.4%). The median time of pneumonia onset was 4.0 days after stroke (IQR, 2–12). A total of 689 pneumonias (64.0%) occurred in the first 7 days after stroke (Figure 1; cumulative incidence of 6.4%). Of all pneumonias in the first 7 days after stroke, the median time of onset was 3.0 days after stroke (IQR, 2.0–4.0). Most pneumonias were diagnosed on the third day after stroke onset (20% of all pneumonias; 30% of all pneumonias in the first 7 days; Figure 2).

Patients with severe stroke (NIHSS score >12) had a pneumonia earlier (median 4.0 days [IQR, 2–12]) than patients with a less severe stroke (NIHSS score ≤12; median 5.0 days [IQR, 3.0–15.8]; *P*=0.02). There were no differences in the day of pneumonia onset between subgroups based on age, sex, stroke type, or treatment with alteplase.

In 1033 patients, a total of 1120 UTIs occurred in the first 90 days after stroke (cumulative incidence 9.5%). The median day of UTI onset was 6.0 days (IQR, 3–14 days), which was later than that of pneumonia (*P*<0.001). In the first 7 days after stroke, 627 UTIs occurred (56% of all UTIs), with the highest incidence on the fourth day (12% of all UTIs; 21% of all UTIs in the first 7 days). The median time of UTI onset in this subgroup was 3.0 days after stroke (IQR, 2–5), which was later than the onset of pneumonia (*P*<0.001).

### Predictors of Pneumonia

Univariate analysis identified higher age, male sex, higher NIHSS, atrial fibrillation, diabetes, coronary heart disease, heart failure, myocardial infarction, and treatment with alteplase as statistically significant predictors of the occurrence of pneumonia in the first 90 days after stroke. In multivariable analysis, higher age, male gender, higher NIHSS, atrial fibrillation, diabetes, and the use of alteplase remained independent predictors (Table 1).

### Outcomes

**Figure 3.**
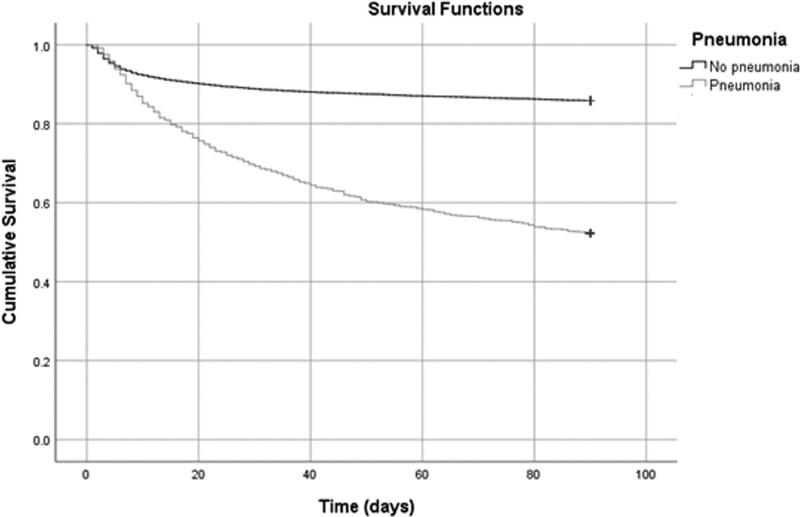
Kaplan-Meier curve showing survival of patients with (dark line) and without (gray line) pneumonia after stroke.

A score on the modified Rankin Scale at 90 days was missing for 241 of the 10 821 patients (2.2%). Of all 1017 patients with pneumonia after stroke, 485 (47.8%) had died at 90 days, compared with 1439 (14.7%) of 9793 patients without pneumonia (*P*<0.001; Figure 3). The median time between the diagnosis of pneumonia and death was 6 days [IQR, 2–18]; 8 days [IQR, 3–22] for pneumonia that started between day 0 and 6, and 3 days [IQR, 1–10] for pneumonia that started between day 7 and 90 (*P*<0.001). The presence of pneumonia was associated with an increased risk of death (adjusted hazard ratio, 4.1 [95% CI, 3.7–4.6]). The association between pneumonia and a higher risk of death remained present in all analyzed time periods (Table 2). A later start of pneumonia appeared to be associated with a higher risk of death, but this was just not statistically significant (aOR, 1.01 [95% CI, 1.0–1.01]; *P*=0.051). Pneumonia in the first 90 days was associated with a poor functional outcome (aOR, 4.8 [95% CI, 3.8–6.1]). The association between pneumonia and poor functional outcome was present in all time periods (Table 2). There was no association between the time of onset of pneumonia and the risk of a poor outcome (aOR, 1.0 [95% CI, 0.99–1.01]; *P*=0.74).

**Table 2.**
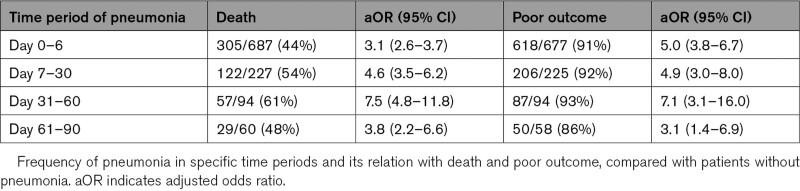
Association Between Timing of Pneumonia and Poor Outcome and Death

## Discussion

The current study based on a large international database confirms earlier reports that about one in every 10 patients has a pneumonia in the first 90 days after stroke and shows that almost 2 out of every 3 pneumonias occur in the first week. The peak incidence of pneumonia was on the third day after stroke, accounting for almost 20% of all pneumonias after stroke. Pneumonias occurred earlier and more frequently in patients with a more severe stroke. The occurrence of pneumonia was independently associated with poor functional outcome or death at any time point during the 90-day follow-up period.

Smaller previous studies reported a median or mean period between stroke onset or hospitalization and pneumonia of 1.8,^8^ 1.9,^16^ 2.0,^17^ 3.0,^9^ or 4.4 days,^10^ observed an peak incidence on the second day,^18^ or found that the majority of pneumonias occurred within 48^7^ or 72 hours.^2,10^ However, these studies were limited by a short follow-up duration (eg, only during hospital admission or limited to the first week or month), reported on small patient numbers (with an amount of pneumonias ranging from 22 to 102), combined all infections,^2,18^ or studied specifically patients in the intensive care unit^8^ or those with dysphagia or tube feeding.^17^ One study evaluated the course of pneumonia after stroke in more detail. This study evaluated 51 respiratory infections in the first 30 days after stroke onset in 369 tube-fed patients and found 2 peaks of infection (on day 1 and on day 3–4).^11^ In contrast to the previous smaller studies, larger cohort studies on infections or pneumonia after stroke did not provide details on the temporal profile,^1,3,5,19,20^ even emphasizing the limited available data on timing.^21^ In our study, we included 10 821 patients with a follow-up duration of 90 days, in whom we observed 1076 pneumonias. The peak incidence occurred between 48 and 72 hours after stroke onset. The cumulative incidence of 9.4% in our study is comparable to the 10% reported in the literature.^1^ Our finding that pneumonia was associated with poor functional outcome or death at any time point during the 90-day follow-up period is in line with previous findings.^12,22^ In addition, we found that where pneumonia occurred >1 week after stroke onset, it resulted in death in fewer days than when pneumonia occurred during the first week after stroke onset (3 versus 8 days). Our finding may reflect more aggressive pneumonia treatment, dysphagia assessment, or more intensive staff input in stroke units compared with more reserved treatment approaches in a later stage after stroke,^23^ for example, because of installed treatment restrictions.

The early peak incidence of pneumonia after stroke in our study is consistent with the 2 most important concepts of the pathophysiology of pneumonia after stroke: stroke-facilitated aspiration and stroke-induced immunodepression.^24,25^ In our study, the median time of pneumonia onset was 4.0 days after stroke. However, the moment of diagnosis is preceded by both an incubation period of the pneumonia as well as the time required for diagnostic evaluation to conclude a diagnosis of pneumonia. Therefore, the temporal profile we found suggests that the process towards the development of most pneumonias starts very early after stroke onset. In addition, pneumonias occurred earlier than UTIs, the second most common infection after stroke. Animal studies have suggested that stroke-induced immunosuppression is already present in the first hours after stroke.^26^ In addition, about 15% of patients with acute ischemic stroke already have signs of pulmonary infection on chest computed tomography imaging within a few hours after stroke onset, suggestive for aspiration at stroke onset or in the first few hours after stroke.^16^ Therefore, the concepts of stroke-facilitated aspiration and stroke-induced immunosuppression are both in accordance with the temporal profile described in the current study.

If preventive antibiotics would be of any benefit, the results of our study suggest that these should be started as soon as possible after stroke and continued for at least 4 days. In the large randomized trials, PASS (Preventive Antibiotics in Stroke Study) and STROKE-INF (Prophylactic Antibiotics After Acute Stroke for Reducing Pneumonia in Patients With Dysphagia), prophylactic antibiotics did not improve outcome after 90 days in patients with acute stroke.^13,14^ PASS had an inclusion window of 24 hours and had a treatment duration of 4 days. There was no association between time to start prophylactic treatment (divided in subgroups of 0–3, 3–6, 6–12, and 12–24 hours) and outcome, but the numbers of patients per subgroup are not known. STROKE-INF had an inclusion window of 48 hours after stroke onset and a treatment duration of 7 days. The study did not report on time-to-treatment subgroup analyses. Both studies found that treatment with antibiotics was safe and associated with just a small number of related adverse events, such as allergic reactions or *C* *difficile* infections. Further insights will be provided by the ongoing PRECIOUS trial (Prevention of Complications to Improve Outcome in Elderly Patients With Acute Stroke), which has a 24-hour inclusion windows and includes elderly patients with moderate to severe stroke, who are, therefore, at the highest risk of pneunomia.^27^ Our findings may also inform (randomized trials on) other prevention strategies, such as dysphagia assessment, swallowing interventions, early mobilization, or oral hygiene improvement.

Our study has limitations. First, there is no uniform definition of pneumonia in the VISTA database. Since the date of onset of the pneumonia was the date that was reported on the adverse event logs, the diagnosis was most likely physician-based rather than an made by an expert panel or on the Centers for Disease Control and Prevention criteria.^28^ Previous studies have shown that the incidence of physician-diagnosed pneumonia is higher than that made by an expert panel.^13,14^ In addition, it is not known which sign or symptom was considered by the local investigator to represent the start of pneumonia. Also, in the VISTA database, several terms are used for reporting respiratory tract infections. We have only selected the terms which include the term pneumonia. Terms that could also include other disease entities were not included (eg, lower respiratory tract infection was excluded because acute bronchitis also falls into this category), so the reported pneumonia incidence could be an underestimation. Second, several factors could have influenced the reporting of pneumonia. Since most data on the occurrence of pneumonia are from SAE reporting, there is a risk that some pneumonias could have occurred before inclusion into the trial or that some pneumonias in trials did not meet the criteria for SAE, and therefore, are not incorporated in the current study. We have tried to minimize this risk by selecting a short inclusion window to start of trial treatment of 24 hours. Also, patients who have a pneumonia at baseline, patients who have either the least or most severe stroke, or patients who have severe comorbidities are less likely to be included in a trial, which could have led to selection bias. Third, we do not have information on (antibiotic) treatment of pneumonia. The treatment type, duration of treatment, and the withholding of treatment could be important to interpret the relation between pneumonia and death or dependency.

In conclusion, pneumonia is a frequent complication in the first 90 days after stroke, with a peak incidence on the third day. Pneumonia was associated with poor functional outcome or death at any time point during the 90-day follow-up period.

## Article Information

### Acknowledgments

Dr de Jonge collected data, performed the analyses, and wrote the first draft of the article. All other authors reviewed the article. All authors read and approved the final version of the article.

### Sources of Funding

The author(s) disclosed receipt of the following financial support for the research, authorship, and publication of this article: Dr de Jonge is funded by the European Union’s Horizon, 2020 research and innovation programme (grant no. 634809).

### Disclosures

Dr van der Worp served as a consultant to Boehringer Ingelheim, Bayer, and LivaNova and received grants from Stryker. Dr van der Worp is Chief Investigator of PRECIOUS (Prevention of Complications to Improve Outcome in Elderly Patients With Acute Stroke), a randomized trial assessing the effects of the prevention of complications in patients with acute ischemic stroke. Dr Bath has served on advisory boards with DiaMedica, Moleac, Nestle, Phagenesis, Platelet Solutions, and Sanofi. Dr Bath is Stroke Association Professor of Stroke Medicine and an Emeritus NIHR Senior Investigator.

### Supplemental Materials

Online Appendices I and II

## APPENDIX

VISTA Steering Committee: K.R. Lees (Chair), A. Alexandrov, P.M. Bath, E. Berge, E. Bluhmki, N. Bornstein, C. Chen, L. Claesson, S.M. Davis, G. Donnan, H.C. Diener, M. Fisher, M. Ginsberg, B. Gregson, J. Grotta, W. Hacke, M.G. Hennerici, M. Hommel, M. Kaste, P. Lyden, J. Marler, K. Muir, N. Venketasubramanian, R. Sacco, A. Shuaib, P. Teal, N.G. Wahlgren, S. Warach, and C. Weimar.

## Supplementary Material


