# Supplementary figures and images for: Temporal Profile of Pneumonia After Stroke
Source: Stroke. 2021 Sep 14;53(1):53–60. doi: 10.1161/STROKEAHA.120.032787 (PMC8700305; doi:10.1161/STROKEAHA.120.032787)

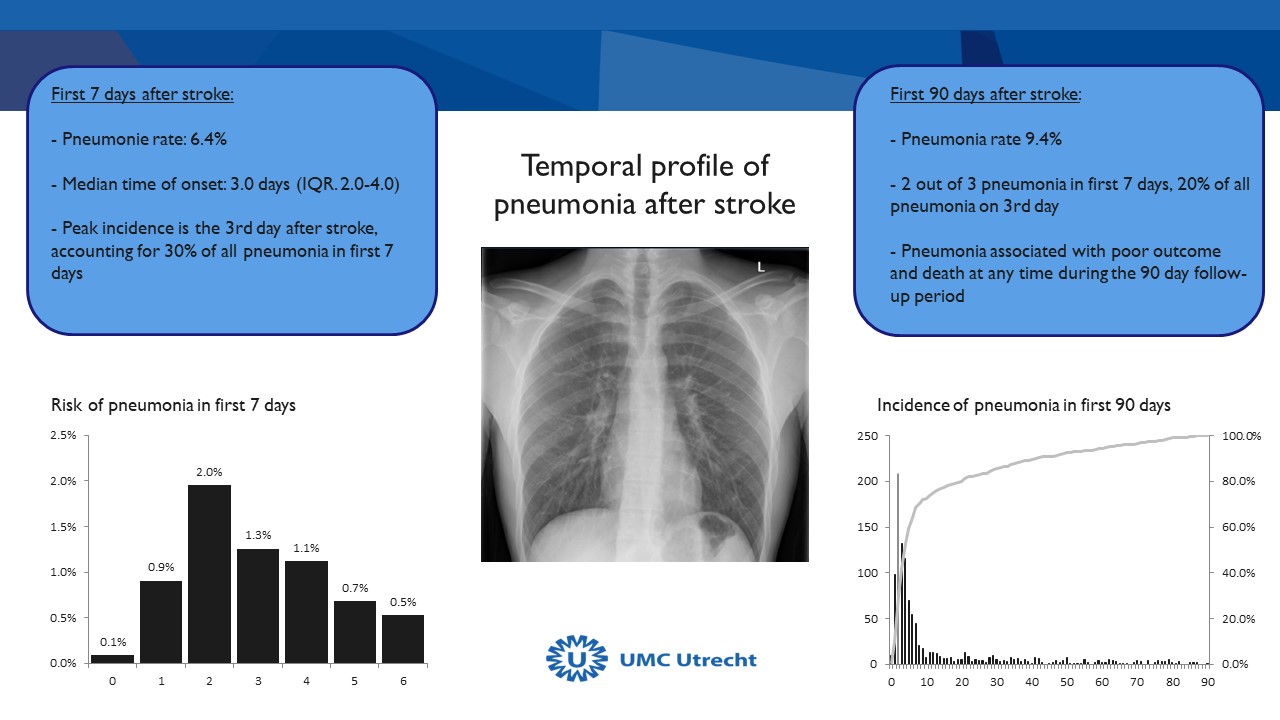

Supplement: Supplementary file 2 [file str-53-053-s002.jpg]
